# Predicting the effect of micro-stimulation on macaque prefrontal activity based on spontaneous circuit dynamics

**DOI:** 10.1103/physrevresearch.5.043211

**Published:** 2024-12-07

**Authors:** Amin Nejatbakhsh, Francesco Fumarola, Saleh Esteki, Taro Toyoizumi, Roozbeh Kiani, Luca Mazzucato

**Affiliations:** 1 Center for Theoretical Neuroscience, Columbia University, New York, New York 10027, USA; 2 Laboratory for Neural Computation and Adaptation, RIKEN Center for Brain Science, Wako, Saitama 351-0198, Japan; 3 Center for Neural Science, New York University, New York, New York 10003, USA; 4 Department of Psychology, New York University, New York, New York 10003, USA; 5 Departments of Biology and Mathematics and Institute of Neuroscience, University of Oregon, Eugene, Oregon 97403, USA

## Abstract

A crucial challenge in targeted manipulation of neural activity is to identify perturbation sites whose stimulation exerts significant effects downstream with high efficacy, a procedure currently achieved by labor-intensive and potentially harmful trial and error. Can one predict the effects of electrical stimulation on neural activity based on the circuit dynamics during spontaneous periods? Here we show that the effects of single-site micro-stimulation on ensemble activity in an alert monkey’s prefrontal cortex can be predicted solely based on the ensemble’s spontaneous activity. We first inferred the ensemble’s causal flow based on the directed functional interactions inferred during spontaneous periods using convergent cross-mapping and showed that it uncovers a causal hierarchy between the recording electrodes. We find that causal flow inferred at rest successfully predicts the spatiotemporal effects of micro-stimulation. We validate the computational features underlying causal flow using ground truth data from recurrent neural network models, showing that it is robust to noise and common inputs. A detailed comparison between convergent-cross mapping and alternative methods based on information theory reveals the advantages of the former method in predicting perturbation effects. Our results elucidate the causal interactions within neural ensembles and will facilitate the design of intervention protocols and targeted circuit manipulations suitable for brain-machine interfaces.

## I. INTRODUCTION

Cognition is an emergent property of collective interactions of neurons in large networks of cortical and subcortical circuits. A promising avenue to ameliorate cognitive function and alter behavior in therapeutic settings is to perform targeted manipulations of brain circuits, which will be greatly facilitated by understanding the causal interactions within these circuits. Successful examples of such manipulations include changing perceived motion direction by altering responses of direction-selective neurons in area MT [1,2], biasing object classification towards faces by altering responses of face-selective neurons in the inferior temporal cortex [3–5], changing the value of a stimulus by altering neural responses in the anterior caudate [6], and controlling movements and postures by altering the activity of motor and premotor cortical neurons [7]. Successful behavioral manipulations depend on the identification of suitable perturbation sites, satisfying at least two requirements. The first is selectivity: the local neural population around the perturbation site should exhibit response properties bearing on the desired behavioral effect, e.g., motion direction selectivity in area MT [1,2], face selectivity in face patches of inferotemporal cortex [3–5], or the locus of seizure in epilepsy [8]. The second requirement is efficacy: stimulation of the local population should exert some significant effect on the activity of the rest of the brain, and consequently on behavior. While selectivity of sensory and motor neurons may be estimated by recording neural activity in simple and well-defined tasks, selectivity tends to be quite complex or variable across tasks in many regions of the association cortex. Discovering efficacy is currently achieved by trial and error: many perturbations are performed until a site whose stimulation leads to a significant change in activity is located. As a result, current methods for targeted perturbations are labor-intensive, time-consuming, potentially harmful, and often unable to generalize beyond the limited task set they are optimized for [9]. Targeted manipulations of brain activity would be greatly improved if one could overcome this trial-and-error procedure and predict the effect of perturbations directly from resting state neural recordings.

Here we address the challenge of predicting the efficacy of a potential perturbation site in the absence of any intervention, by inferring the causal flow (CF) between recording electrodes within the local neural circuit during spontaneous periods. Intuitively, one expects that perturbing a node with strong CF to other nodes within a circuit may exert stronger effects than perturbing the nodes that are functionally isolated. Our aim is to first infer the CF from sparse recordings of spontaneous spiking activity, obtained from chronically implanted prefrontal multi-electrode arrays in awake, resting monkeys. Using the CF between electrodes inferred during resting activity, we then aim to predict the effect of electrical micro-stimulations of single electrodes on the rest of the circuit. We characterize the effects of perturbations by introducing the concept of interventional connectivity (IC), an observable that is agnostic to the underlying structural connectivity and depends on only responses to perturbations.

Estimating the functional connectivity between cortical circuits is a central open problem in neuroscience [10], which is challenged by the very specific features of cortical dynamics, such as strong common inputs from unobserved brain areas, weak correlations, complex nonlinear interactions, and extremely sparse sampling (i.e., from a small fraction of neurons in a circuit). We infer the CF between recording electrodes using data-driven time series forecasting (specifically, convergent cross-mapping [11]), a method from the theory of deterministic dynamical systems, which aims to reconstruct the network attractor in a high-dimensional state space obtained using a delay embedding approach. This method was expressly developed to work in the sparse data regime [12,13] and in the presence of noise, common inputs, and nonlinear couplings between variables [11]. While this powerful framework, rigorously articulated in [14], has been successfully applied in ecology [11], and *in vitro* [11,15] and ECoG neural activity [16], it has never been adapted to spiking activity *in vivo*.

We perform a series of simulated experiments based on recurrent neural networks to determine the applicability regimes of CF and its predictive ability. In these simulated experiments, we show that this method can overcome complex challenges, such as noise and common inputs. These simulations validate the accuracy and efficiency of the CF estimation method. We then performed a detailed comparison between CF inference based on convergent cross-mapping (CCM) with several alternative methods commonly used to infer functional interactions, including information-based methods such as Granger causality and transfer entropy. This critical comparison demonstrates the superior performance of the CCM-based CF method in predicting micro-stimulation effects in the alert monkey prefrontal cortex. We have created and shared an open-source tutorial software package for the estimation of CF from CCM and information-based measures on simulated and empirical data, which can be efficiently and scalably run on standard computers [17]. In summary, our results highlight the advantages of deploying CF to guide perturbation experiments compared to traditional methods, opening the way towards efficient protocols for targeted manipulations of cortical ensembles in primates and humans.

## II. RESULTS

*Notation:* Throughout the paper, we use lower-case letters for scalars and scalar-valued functions [e.g., *τ, x*_*i*_(*t*)], bold letters for vectors (e.g., ***f*** , ***X***), and calligraphic letters for sets (e.g., 𝒳). We use the terms *electrode* and *cluster* interchangeably as every electrode is located in the vicinity of a cluster of neurons.

### A. Spatiotemporal features of microstimulation on cortical circuits in alert monkeys

**FIG. 1.**
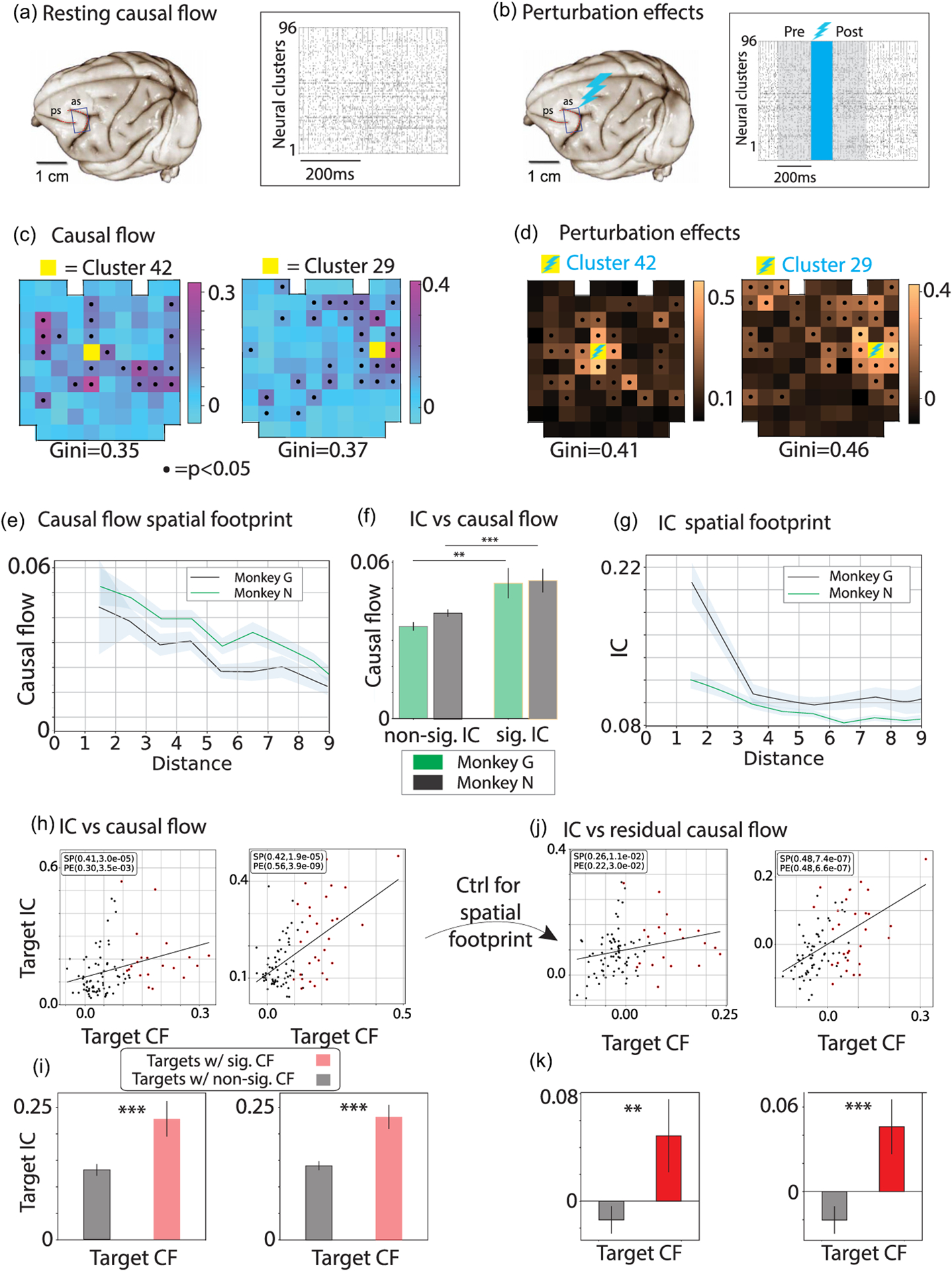
CF inferred at rest predicts micro-stimulation effects in alert monkeys. (a) Ensemble spiking activity of pre-arcuate gyrus recorded with a 96-channel multi-electrode array. Black tick marks are spikes, and each row shows the aggregated spike train of the neural cluster recorded by each electrode. (b) Example trial showing that micro-stimulation of electrode 15 (a 120 ms pulse train with biphasic 15 μA stimulation pulses, blue shaded area) perturbs activity across neural clusters. (c) CF vectors for two representative source clusters 42 and 29. Yellow squares show the location of the source cluster on the array. CF vectors for each source are also overlaid on the array geometry (black dots represent significant CF values, established by comparison with surrogate data sets, *p* < 0.05; see Fig. 2 and Supplemental Material [29]). For the full CF matrix see Fig. S8 [29]. (d) IC for the representative stimulated clusters 42 and 29 in monkey N, measured based on spike count distributions in 200 ms intervals preceding and following the stimulation [gray shading in the population raster plot; black dots represent significant ICs (*p* < 0.05); yellow squares show the location of the stimulated cluster]. (e) Spatial footprint of CF as a function of target channel distance from source channel [here and in panels below, shaded area or error bar represents s.e.m. across seven sessions in monkey G (black) and seven sessions in monkey N (green)]. (f) Average CF for source-target pairs with significant (right) and nonsignificant (left) IC. (g) Spatial footprint of the perturbation effects calculated as a function of target electrode distance from the stimulated electrode. (h) Resting state CF predicts perturbation effects. For each stimulated source, target clusters with larger CF are more strongly impacted by micro-stimulation, indicated by larger ICs. Gray and red data points represent targets with nonsignificant and significant CF, respectively (*p* < 0.05). Black lines are linear regressions. *R*^2^ and slope *p* value are reported. (i) For each stimulated source, aggregated perturbation effects are larger over targets with significant CF vs targets with nonsignificant CF. Error bars are s.e.m. *, **, *** indicate *p* < 0.05, 0.01, 0.001 (*t* test). (j), (k) Controlling for spatial dependence of CF and perturbation effects. The residual CF and perturbation effects are from (a) and (c). Residuals are calculated by removing the dependence on distance from the source electrode (see Supplemental Material for details). (j) Correlation between CF and IC after controlling for spatial dependence. (k) Mean residual IC for target clusters with significant and nonsignificant CF for each source electrode.

To test whether intervention effects can be predicted from the spontaneous activity of neurons, we recorded from and micro-stimulated the monkey prefrontal cortex (pre-arcuate gyrus, area 8Ar) during a period of quiet wakefulness (resting) while the animals were sitting awake in the dark. The experiment had two phases [Figs. 1(a) and 1(b)]. In the first phase [Fig. 1(a)], we recorded population neural activity from a multi-electrode array (96-channel Utah array, with roughly one electrode in each cortical column in a 4 × 4 mm^2^ area of the cortex), estimating the CF between pairs of neural clusters (multiunit activities collected by each recording electrode). The goal of the first phase is to infer the CF between recording electrodes at rest [Fig. 1(c)]. In the second phase [Fig. 1(b)], we perturbed cortical responses by delivering a train of biphasic micro-stimulating pulses (15 μ*A*, 200 Hz) to one of the clusters for a brief period (120 ms), recording population neural activity across the array before and after the stimulation. We calibrated the electrical micro-stimulation to be short and weak, substantially below the threshold for evoking eye movements [18]. The goal of the second phase is to estimate the effects of micro-stimulation [Fig. 1(d)].

To characterize the effect of micro-stimulation on the cortical activity, we measured the dissimilarity of the neural activity distribution in the intervals preceding the onset and following the offset of the stimulation for each pair of stimulated source and recorded target electrode [see Fig. 1(d)]. We focused on the activity after offset as opposed to during the stimulation period to minimize the effect of stimulation artifacts on the recording apparatus. The overall effect of stimulating source electrode *j* on the neural activity recorded by target electrode *i* could then be determined as a *perturbation vector*, $s^{\left(j\right)}={\left\{S_{ij}\right\}}_{i=1}^{N}$, where *S*_*i j*_ is the IC between target unit *I* and source unit *j*. IC is quantified as the Kolmogorov-Smirnov statistics between pre- and post-stimulation spiking activity distributions over stimulated trials (see Supplemental Material for details). Because each electrode records from a cluster of neurons around the electrode tip, the perturbation vector quantifies changes in the activity of spatially distinct neural clusters across the recording array.

We first examined the spatiotemporal features of stimulation effects. We found that even short, subthreshold perturbations exerted a strong effect on ensemble activity and that these effects were specific to which electrode was stimulated [Fig. 1(d); perturbation effects for representative stimulated source electrode *j* are visualized as a perturbation vector ***s***^(*j*)^ overlaid on the array geometry]. Two observations stand out. First, we found a clear spatial gradient in perturbation effects, with an overall decreasing effect for target clusters farther from the source electrode [Fig. 1(g)]. However, some distant targets were also strongly affected. The identity of strongly modulated targets was specific to the stimulating electrode, exhibiting an inhomogeneous circuit structure, also apparent in the Gini coefficients of the perturbation vectors, showing large values [Fig. 1(d)].

The Gini coefficient quantifies the hierarchical structure in IC vectors by estimating the degree of uniformness in their distribution. For example, a delta distribution where all samples have the same value has zero Gini coefficient, while an exponential distribution has a Gini coefficient equal to 0.5. In the absence of hierarchical structures, one would expect all targets from a given source unit to have comparable IC values, yielding a low Gini coefficient. Alternatively, heterogeneity of IC values across targets for a given source would suggest a network hierarchy with a gradient of connectivities, yielding a large Gini coefficient.

### B. Inferring CF from resting blocks in alert monkeys

**FIG. 2.**
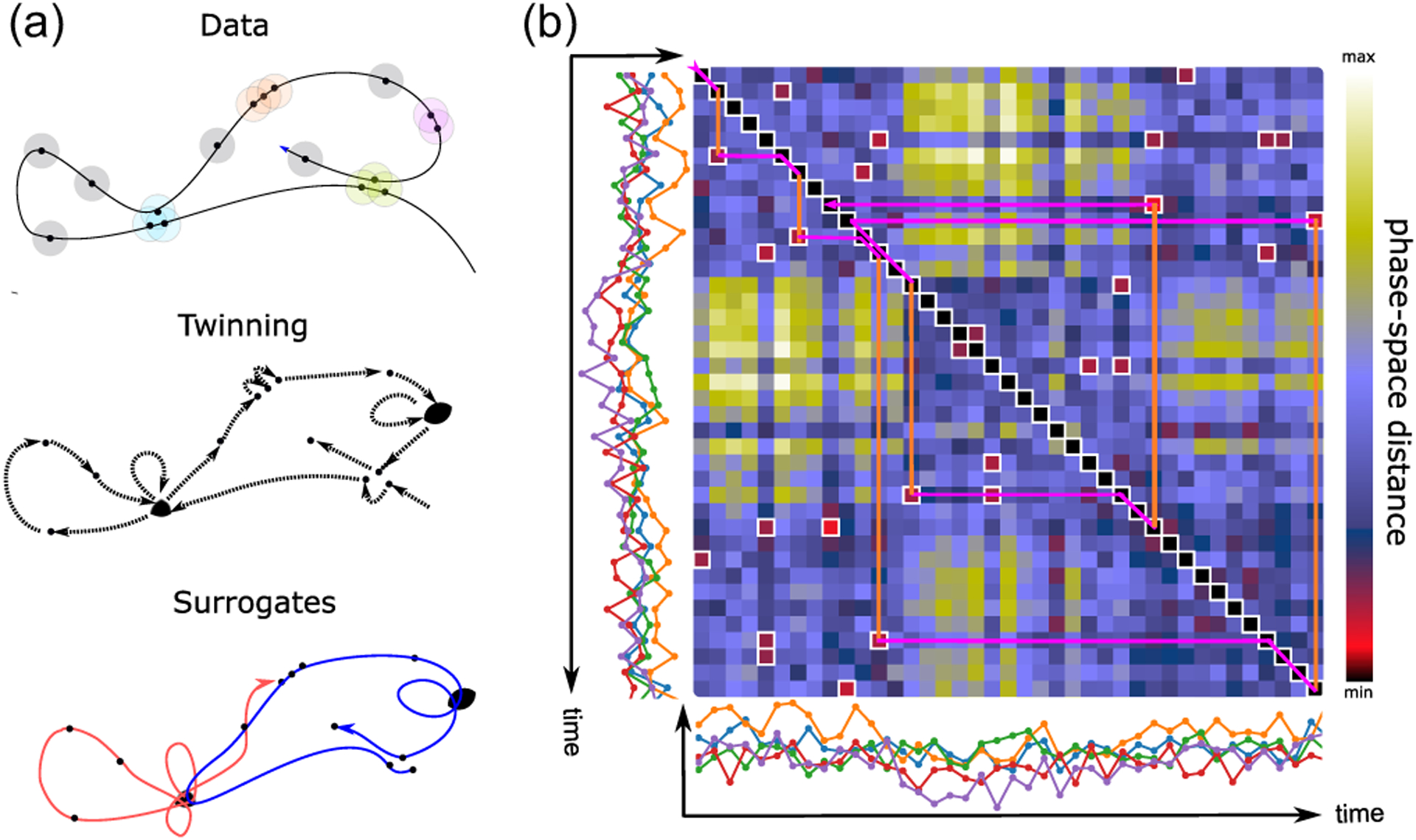
Establishing significance of CF. (a) The significance of CF is determined by comparison to surrogate data sets designed to preserve all large-scale nonlinear properties of the system. Surrogates are produced in three stages: top, phase-space distance is evaluated among Takens states constructed from each time series, and nearest neighbors are identified; center, states in the trajectory are coarse-grained by collapsing states with the same set of neighbors (in the example, the blue and purple clusters merge but the orange and green ones do not); bottom, surrogate trajectories are generated from random initial conditions by regarding twin sets as retentive states in a Markov process [retention is represented by self-loop and has probability *p* = (*n* − 1)*/n* where *n* is the number of twins]. (b) Example trajectory depicted over the matrix of phase-space distances for the multidimensional time series shown along both axes. Whereas the main diagonal corresponds to the flow of the recorded time series, at each step the surrogate time series can either move forward as in the recording or depart from the main diagonal (vertical orange lines) to pick one of the low-distance states in its own twin set, and perform from there a forward step mimicking the recording (purple broken lines). Notice that, as in the example, motion can be both forward and backward in time.

Can we predict the effects of stimulation solely based on features of neural activity estimated during resting blocks, in the absence of stimulation? To answer this question, we first inferred the directed causal functional connectivity from the source electrode *j* to each one of the target electrodes during resting periods, which we refer to as CF. We defined the CF vector as the *N*-dimensional vector $f^{\left(j\right)}={\left\{F_{ij}\right\}}_{i=1}^{N}$, representing the directed functional connectivity from the source electrode *j* to each one of the target electrodes *i*. We inferred the CF using convergent cross-mapping (CCM) [11], a method developed for deterministic dynamical systems aiming at inferring the ensemble’s dynamical attractor in a high-dimensional state space obtained via delay embedding (see Table S3 for details). We proceeded to infer CF using CCM by reconstructing the temporal series of a source electrode *x*_*i*_ (*t*) given a target electrode *x*_*j*_ (*t*) from the corresponding delay vectors

(1) $$X_{i}(t)=\left[x_{i}(t),x_{i}(t-\tau ),\ldots ,x_{i}(t-d\tau +\tau )\right]$$

of dimension *d* with step size *τ*. Reconstruction accuracy was quantified as the Fisher *z* transform *z***(***ρ*(***X****i*|***X***_*j*_)**)** of the Pearson correlation *ρ*(***X***_*i*_|***X***_*j*_) between the *empirical* activity of the delay vector of electrode *i* and its *predicted* activity obtained from the delay vector of electrode *j*. Whereas the Pearson correlation is bounded between −1 and 1, its Fisher *z* transform is approximately normally distributed thus facilitating statistical comparisons [19]. The process was cross-validated to avoid overfitting [see Methods for details; model selection for hyperparameters *d* (delay dimension) and *τ* (time step) is reported in Fig. S7]. We established statistical significance by comparing the CF estimated from the empirical data with that estimated from surrogate data sets designed to preserve the temporal statistics of the network activity while breaking its causal structure [20] (see Fig. 2 and Supplemental Material for details). Columns of the CF represent the *source* electrode, whose activity is being re-constructed, and rows represent the *target* electrode, whose activity is used for the reconstruction.

The CF inferred at rest was characterized by a set of spatiotemporal features (for the full 96 × 96 CF matrix see Fig. S8; see Methods for details and for model selection). In Fig. 1(c) we show four representative 96-dimensional CF vectors representing the CF for two different source clusters (channels 42 and 29 from monkey N; these source clusters were later micro-stimulated in the second phase of those sessions). The CF vectors are overlaid onto the array geometry (location of recording electrodes in the array) for illustration [each overlaid CF vector in Fig. 1(c) corresponds to a specific column of the full CF matrix in Fig. S8 [29]]. Comparison of the CF vectors across source clusters revealed remarkable features about the structure of the functional connectivity. First, CF is strongly channel-specific, namely, it depends on the source clusters whose activity is being reconstructed. Second, each CF vector shows a hierarchical structure, with significant CFs for a small subset of targets, while most targets cannot reconstruct the source activity [Fig. 1(c)], confirmed by the high values of their Gini coefficients. This result is qualitatively consistent with the hypothesis of functional hierarchies embedded within prefrontal cortical circuits [21].

### C. CF predicts microstimulation effects in alert monkeys

Our hypothesis posits that the effects of stimulation of source cluster *j* on the target neural clusters can be predicted by the corresponding CF vector ***f***^(*j*)^ inferred at rest (i.e., a column of the CF matrix). Specifically, we predict that those target clusters that have a stronger CF to the source as defined by the source CF vector will exhibit stronger perturbation effects following stimulation of the source. Visual inspection of the resting state CF vectors [Fig. 1(c)] and the map of perturbation effects [Fig. 1(d), perturbation vectors] suggests channel-specific similarities between the two observables, in particular, a strong resemblance between the spatial pattern of CF and of stimulation [Fig. 1(e)]. A quantitative comparison with the spatial footprint of micro-stimulation [Fig. 1(g)] revealed that CF captured the spatially decaying profile of the micro-stimulation. Moreover, source-target pairs exhibiting a significant micro-stimulation effect had a significantly larger CF, compared to the pairs exhibiting no stimulation effects [Fig. 1(f)], a feature consistent across both monkeys with a large effect size.

To further examine the predictive power of CF, we explored if statistically significant CF indicated larger perturbation effects. We developed a method to establish the significance of CF pairs based on surrogate data sets created from the so-called “twin surrogates” (Fig. 2).

The twin-surrogate method was first proposed in [20], which was designed to preserve all large-scale nonlinear properties of the system. Surrogate time series are produced in three stages. First, we evaluated phase-space distances among Takens states constructed from each time series: nearest neighbors were defined as states closer to each other than some neighborhood radius. Second, an equivalence relationship was defined between states possessing the same set of neighbors (known as “twins”). Finally, surrogate trajectories were initialized randomly and generated by allowing each subsequent step to start with equal probability from its current state or from one of its twins. See Sec. A 4 for technical details.

We then tested whether stimulation of a given source cluster led to a larger perturbation effect in target clusters with a significant CF compared to the target channels with no significant CF with the source cluster. Indeed, we found that to be the case, as predicted by our theory [Figs. 1(h) and 1(i)]. The predictive power of CF is held at the level of single stimulated source electrodes, thus achieving a high level of granularity in prediction. Because both the CF and perturbation magnitude displayed a characteristic decay proportional to the distance from the source electrode, we tested whether the predictive relationship between the CF and perturbation magnitude might have been solely shaped by spatial distance. After removing this spatial dependence, the predictive relation still held between the residual CF and perturbation effects [Figs. 1(j) and 1(k)], confirming that the CF predicts perturbation effects above and beyond what is expected from a spatial decay away from the source electrode (see Supplemental Material for details).

These results demonstrate that the CF estimated from sparse recordings during the resting periods accurately predicts the effects of perturbation on the neural ensemble, thus establishing the validity of our hypothesis in cortical circuits of alert monkeys.

### D. CF validation using recurrent neural networks

Our results showed that CF inferred from resting activity can accurately predict micro-stimulation effects. To explain the theoretical underpinnings of this success and to probe its regime and range of validity, we developed a series of analyses based on ground-truth synthetic data. We simulated a continuous rate network comprising both feed-forward and recurrent features in its structural connectivity, where we arbitrarily varied the noise levels and features to assess robustness against changes in signal-to-noise ratios and common inputs. The goal of this simulated experiment was to test the extent to which CF can infer a circuit’s hierarchical causality structure and provide validation for our method with ground-truth synthetic data.

We first simulated a deterministic network, comprising *N* units arranged in two subnetworks 𝒳 and 𝒴, each endowed with their own local recurrent connectivity. Crucially, there are directed projections from units in 𝒳 to units in 𝒴 with coupling strength *g*, but no feedback couplings from units in 𝒴 to units in . Specifically, the network consists of 100 + 3 nodes, three of which belong to the subnetwork 𝒳 following Rössler dynamics with strong all-to-all recurrent described by the equations below:

$$\tau_{0}\frac{dx_{1}}{dt}=-x_{2}-x_{3},\;\tau_{0}\frac{dx_{2}}{dt}=x_{1}+\alpha x_{2},\;\tau_{0}\frac{dx_{3}}{dt}=\beta +x_{3}\left(x_{1}-\gamma \right).$$

**FIG. 3.**
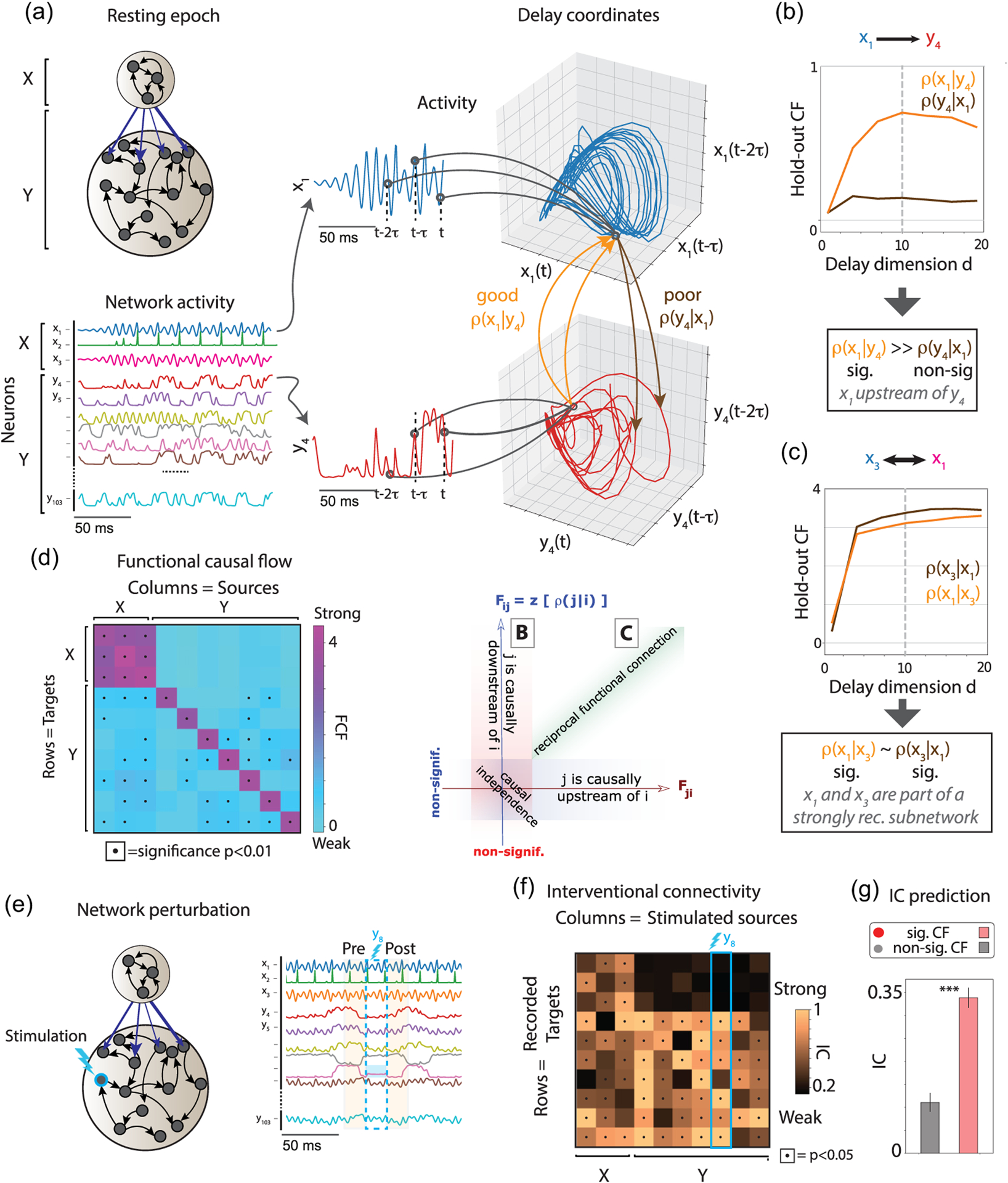
CF validation in recurrent networks. (a) Left: Schematic of network architecture: two subnetworks 𝒳 (top nodes) and 𝒴 (bottom nodes) with strong and weak recurrent couplings within the subnetworks, respectively, are connected via feed-forward couplings from 𝒳 to 𝒴 The thickness of the black arrows represents the strength of directed structural couplings. Center: Activity of units *y*_4_(*t*) (red, bottom) and *x*_1_(*Yt*) (blue, top) are mapped to the delay coordinate space ***X***_1_ = [*x*_1_(*t*)*, x*_1_(*t* − *τ*), …*, x*_1_**(***t* − (*d* − 1)*τ***)**] and ***Y***_4_ [right, *τ =* 4 ms; see Fig S7(A)]. (b) Reconstruction accuracy increases with delay vector dimension *d* before plateauing. The reconstruction accuracy *ρ*(***X***_1_|***Y***_4_) of upstream unit *x*_1_ given the downstream unit *y*_4_ is significant and larger than the reconstruction accuracy *ρ*(***Y***_4_|***X***_1_) of *y*_4_ given *x*_1_ (nonsignificant). The CF value *F*_41_ reveals a strong and significant CF from upstream node *x*_1_ to downstream node *y*_4_ . (c) The significant CF between two units *x*_1_ and *x*_3_ within the strongly coupled subnetwork 𝒳 reveals strong and significant CF between them, but no preferred directionality of CF. (d) Left: The CF between 10 representative units sparsely sampled from the network (columns and rows represent source and target units, respectively; columns are sorted from functionally upstream to downstream units). Right: Summary of the CF cases in panels (a)–(c) (see Table S3 [29]). (e) CF predicts perturbation effects. Perturbation protocol: single nodes (e.g., unit *y*_8_) are stimulated with a pulse of strength *S* lasting for 100 ms. Perturbation effects on target units are estimated by comparing the activity immediately preceding the onset and following the offset of stimulation to calculate IC. (f) IC matrix for the same units in (d). Black dots represent significant effects (*p* < 0.05). The effects of stimulating one source *j* on all targets are encoded in the perturbation vector ***s***^(*j*)^. (g) target units with significant resting-state CF had a larger IC, compared to target units with the nonsignificant CF (*t* test, *** indicates *p* < 10^−6^).

Nodes in subnetwork 𝒴 have weak recurrent couplings and evolve according to the following dynamics:

$$\tau_{0}\frac{dy}{dt}=-\lambda y+10\;\tanh\left(J_{𝒴𝒳}x+J_{𝒴𝒴y}\right)+I,$$

where ***x*** = *x*_1:3_, ***y*** = *y*_4:103_. The weight matrix *J*_𝒴𝒳_ connecting 𝒳 to 𝒴 is the product of a scalar *g* (connection strength) and a matrix of all ones. The recurrent weight matrix *J*_𝒴𝒴_ is drawn from 𝒩 (0*, g*_*r*_). Increasing *g*_*r*_ will transition the network into a chaotic regime. See Table S1 for a description of the model parameters and their values. Using this model, we aimed to capture the intuitive idea that the upstream subnetwork 𝒳 drives the activity of the downstream subnetwork 𝒴 (Fig. 3).

We inferred the CF between all pairs of units in the network. Figure 3(b) shows the CF between a unit in the downstream subnetwork 𝒴 (i.e., *y*_4_) and a unit in the upstream subnetwork 𝒳 (i.e., *x*_1_). The activity of unit *x*_1_ depends on only the other units in , to which it is recurrently connected, but not on the units in 𝒴, as there are no feedback couplings from 𝒴 to 𝒳 . On the other hand, unit *y*_4_ receives direct projections from unit *x*_1_ and is causally influenced by it. Thus, we expect that the reconstruction of *x*_1_(*t*) from *y*_4_(*t*) will be more accurate than the reconstruction of *y*_4_(*t*) from *x*_1_(*t*). As expected, the cross-validated reconstruction accuracy increased as a function of the Takens dimension *d* of the delay coordinate vector [Figs. 3(b) and 3(c)]. The accuracy plateaued beyond a certain dimensionality (related to the complexity of the time series [15]), whose value we chose for our subsequent analyses (see Fig. S7(A) for hyperparameters *d* and *τ* model selection).

In the example above, the reconstruction accuracy of *x*_*j*_ given *y*_*i*_ was significant and large, while that of *y*_*i*_ given *x*_*j*_ was not significant. In other words, while one can significantly reconstruct *x*_*j*_ with high accuracy from *y*_*i*_, because the latter receives information from the former, the opposite is not possible, matching predictions based on the simulated network architecture. We refer to *x*_*j*_ as being *causally upstream* to *y*_*i*_ in the CF of the network.

The variety of CF features discussed so far suggests that even though the CF is a measure of pairwise causal interactions, it may reveal a network’s global causal structure. A principal component analysis of the CF vectors ***f***^(*j*)^ from a sparse subsample of the network units (10 out of 103) revealed a clear hierarchical structure present in the network dynamics showing two separate clusters corresponding to the subnetworks 𝒳 and 𝒴 [Fig. S9(A) [29]]. Thus, CF vectors revealed the global network functional hierarchy from sparse recordings of the activity, which was confirmed using alternative methods such as the Gini coefficient of CF vectors [Fig. S9(B) [29]].

### E. Validating the predictive power of CF in recurrent neural networks

Can we predict the effects of perturbations on network activity based on the CF inferred from the unperturbed system? We hypothesized that the effects of stimulating a specific node on the other nodes of a network can be predicted by the CF inferred during the resting periods.

We simulated a perturbation protocol where we artificially imposed an external input on one source unit for a brief duration, mimicking electrical or optical stimulation protocols of cortical circuits, and estimated the stimulation effect using IC [Fig. 3(e)]. We found that stimulation exerted complex spatiotemporal patterns of response across the target units, captured by the perturbation vectors. In our simulations, stimulation effects across targets *k* strongly depended on the source unit *j* that was stimulated. Perturbation effects increased with stimulation strength for source-target pairs in 𝒳 → 𝒳 𝒳 → 𝒴 and 𝒴 → 𝒴, but not for pairs , 𝒴 → 𝒳 consistent with the underlying structural connectivity lacking feedback couplings from 𝒴 to 𝒳 [Fig. S9(C)]. Can one predict the complex spatiotemporal effects of stimulation solely based on the CF inferred during resting activity?

We hypothesized that, when manipulating source unit *j*, its effect on target unit *i* could be predicted by the CF estimated in the absence of perturbation [Fig. 3(g)]. Specifically, we tested whether stimulation of source unit *j* would exert effects only on those target units *i* that have significant CFs, *F*_*ij*_, but no effects on units whose CFs were not significant. We found that the perturbation effects on the target units were localized to units with significant CFs [dots in Fig. 3(d); Fig. 3(f)]. No effects were detected on pairs with nonsignificant CF. In particular, we found that pairs where the stimulated source was in 𝒴 and the target in 𝒳 did not show any significant effects of perturbations [Fig. 3(g)]; this was expected given the absence of feedback couplings 𝒴 → 𝒳. Conversely, we found that source-target pairs with significant IC after perturbation had much larger resting state CF compared to pairs with nonsignificant IC [Fig. 3(g)]. Two crucial features of the CF, underlying its predictive power, were its directed structure and its causal properties. We thus conclude that the causal effect of perturbations on network units can be reliably and robustly predicted by the CF inferred during the resting periods (i.e., in the absence of the perturbation).

### F. Robustness of CF estimation to noise and common inputs

Neural circuits *in vivo* include multiple sources of variability including both private (e.g., Poisson variability in spike times) and shared variability (e.g., low-rank co-fluctuations across the neural ensemble [22,23]), where the latter may correlate to the animal’s internal state such as attention or arousal [24–27]. Shared sources of external variability represent common inputs, which notoriously pose strong challenges to existing methods to infer functional connectivity. Based on previous theoretical work, we expected our CF framework to be reasonably resilient against these effects [28].

**FIG. 4.**
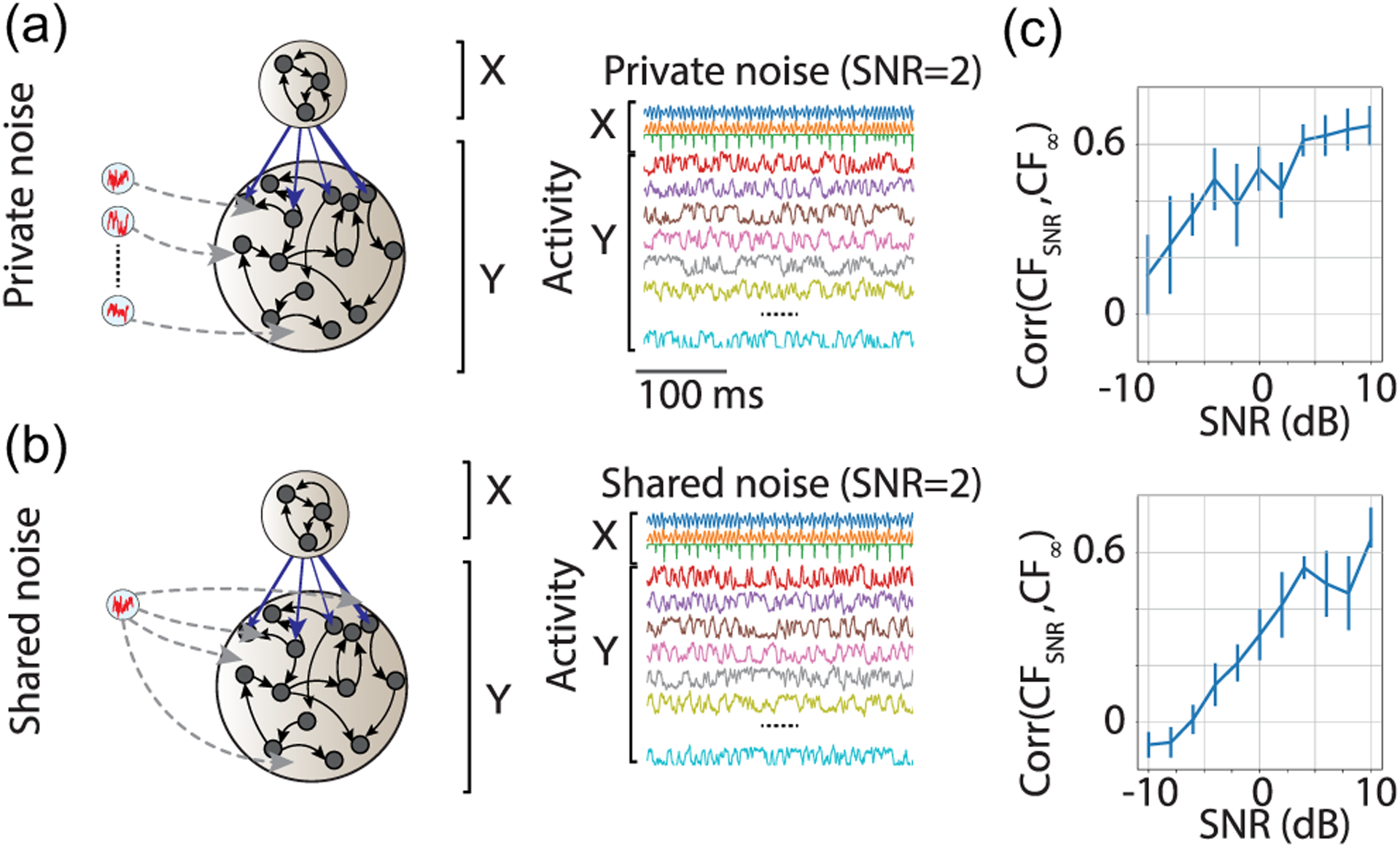
Robustness of inferred CFs to time-dependent inputs. CF is robust to both private noise (a, i.i.d. realizations of noise in each unit) or shared noise (b, a scalar noise source modulates all neurons). The same network as in Fig. 3. (c) Correlation of CF inferred from the noiseless simulations (CF_∞_) with the CF inferred from simulations with varying SNR (SNR, defined in units of dB as 10 log_10_[*σ* (signal)*/σ* (noise)], where *σ* is standard deviation). Top: private noise; bottom: shared noise.

To quantify the robustness of inferred CFs, we tested their changes as a function of the strength of a noisy input to subnetwork 𝒴. When driving the network with either private noise [i.i.d. for each neuron; Fig. 4(a)] or common input [shared noise, namely, the same noise realizations across all neurons; Fig. 4(b)], we found that CF inference degraded only when the signal-to-noise ratio (SNR) dropped below 0 dB [Fig. 4(c); SNR is measured in logarithmic scale]. However, for a wide range of SNRs, the CF inference maintained its accuracy. The degradation caused by private noise did not remove the informativeness of CFs for the tested range of SNRs down to −10 dB. Common external input in the form of shared noise became irrecoverably detrimental only for SNRs below −5. We thus conclude that CF estimates are robust to both noise and external time-varying inputs, two common sources of neural variability.

Our validation results thus demonstrate that the data-driven discovery of CF is robust to noise.

### G. Comparing CF predictions with alternative methods

**FIG. 5.**
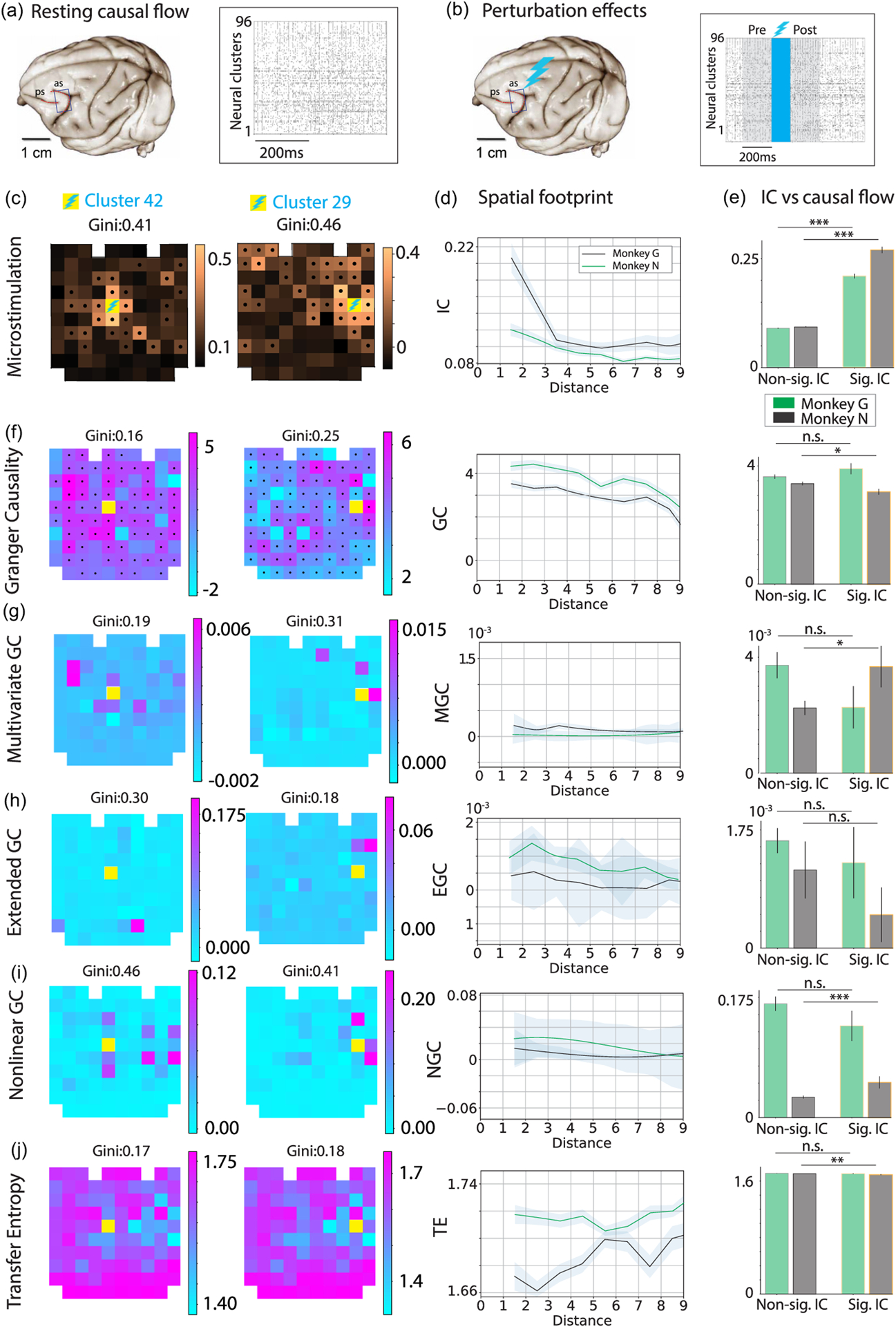
Stimulation of a source electrode evokes a complex, hierarchical, and electrode-specific pattern of perturbations across the circuit that is not captured by existing information-based causality measures. (a) Ensemble spiking activity of pre-arcuate gyrus recorded with a 96-channel multi-electrode array. Black tick marks are spikes and each row shows the aggregated spike train of the neural cluster recorded by each electrode. (b) Example trial showing that micro-stimulation of electrode 15 (a 120 ms pulse train with biphasic 15 μA stimulation pulses, blue shaded area) perturbs activity across neural clusters. (c) IC for the representative stimulated clusters 42 and 29 in monkey N, measured based on spike count distributions in 200 ms intervals preceding and following the stimulation (gray shading in the population raster plot; black dots represent significant ICs (*p* < 0.05); yellow squares show the location of the stimulated cluster). (d) Spatial footprint of the perturbation effects calculated as a function of target electrode distance from the stimulated electrode. (e) Average IC values for source-target pairs with significant (right) and nonsignificant (left) IC. (f)–(j) Information-based estimates of CF using Granger causality (GC, f), multivariate GC (g), extended GC (h), nonlinear GC (i), and transfer entropy (TE, j). First two columns: Causal vectors for two representative source clusters (yellow squares show the location of the source cluster). Causal vectors for each source are also overlaid on the array geometry. Third column: spatial footprint of the causal vectors estimated as in (d). Fourth column: Average CF measures for source-target pairs with significant (right) and nonsignificant (left) IC. * = *p* < 0.05, ** = *p* < 0.01, *** = *p* < 0.001 (*t*-test). Shaded areas or error bars represent s.e.m. across seven sessions in monkey G (black) and seven sessions in monkey N (green).

We performed a critical comparison of our CF approach with several alternative methods for estimating directed functional connectivity based on information theory. Within the class of information-based methods, we estimated functional connectivity vectors during the resting state using traditional causality measures based on information transfer [Figs. 5(e)–5(i)], specifically: Granger causality in its univariate (GC) and multivariate (MGC) formulations; nonlinear GC (NGC) with radial basis functions for autoregression; extended GC (EGC); and transfer entropy (TE). Information-based methods assume that ensemble neural activity is a *stochastic process* and aim to test whether the uncertainty in estimating the future activity $x_{j}^{\text{future}}$ of the target electrode *i* based on its past activity $x_{i}^{\text{past}}$ can be improved by including the past activity $x_{j}^{\text{past}}$ of the source electrode *j*. A significant reduction in uncertainty leads to a positive value of directed functional connectivity from *j* to *i*. While TE is based on the nonparametric comparison, GC and its variations make the further assumption that the activity can be modeled as a Gaussian autoregressive process (we refer the reader to the Supplemental Material [29] Sec. B 1 for a detailed examination of the technical and conceptual differences between information-based and CCM-based approaches). For our comparisons, we first inferred the functional connectivity from resting blocks using each alternative method and then examined how well each of these functional connectivity estimates could predict the effects of stimulation.

We first performed this comparison on ground-truth synthetic data generated from recurrent neural networks, following the same experimental protocol and analysis as we did for CF in Fig. 1. We found that CCM-based CF had a significantly larger positive correlation with IC (*r =* 0.50, *p* = 1.7 × 10^−6^) compared to all other methods (Fig. S11). Among the other methods, only GC had a significant positive correlation as well (*r* = 0.32, *p* = 2.8 × 10^−3^). These results were corroborated by an additional test that showed source-target pairs with significant IC had larger CCM-based CF (*p* = 1.8 × 10^−9^) and GC (*p =* 9 × 10^−5^) than pairs with nonsignificant IC, whereas the other methods did not exhibit such a difference. From these comparative analyses on simulated data we concluded that, among the causality methods, CF performs best. GC is also predictive of perturbation effects, although to a lesser degree than CF, while NGC, TE, and MGC do not exhibit predictive features.

We then performed this comparison on data from alert monkeys, following the same protocol as we did for CF in Fig. 1. We based this comparison on two metrics: spatial footprint and perturbation vectors [Figs. 5(e)–5(i)]. We first estimated the predictive ability of each information-based method. We found that these methods were not able to capture the spatial footprint of the perturbation effects, which featured a monotonic decay with distance from the stimulated electrode [Fig. 5(d)], except for GC, which exhibited a monotonically decaying spatial dependence. We then tested the relationship between perturbation vectors and CF vectors estimated from information-based methods at a more granular level. We tested whether source-target pairs with significant stimulation effects had a significantly larger functional connectivity measure compared to source-target pairs without significant stimulation effects. None of the information-based metrics exhibited large functional connectivity for pairs with significant IC compared to pairs with nonsignificant IC in both monkeys [Figs. 5(f)–5(j)]. In particular, these methods yielded inconsistent estimates of CF between the two monkeys. We concluded that, although overall the information-based methods for estimating causality were capable of capturing some of the electrode-specific stimulation effects, and GC in particular yielded promising results on synthetic data, none of them significantly captured both the spatial footprint of perturbation and the more granular prediction of perturbation vectors in alert monkeys.

## III. DISCUSSION

We demonstrated a framework for predicting the effect of perturbations on a cortical circuit based on the causal interactions within a circuit inferred from sparsely recorded spiking activity at rest. We found that the effects of single-site micro-stimulation on ensemble activity in an alert monkey’s prefrontal cortex can be predicted solely based on the causal flow inferred from ensemble spontaneous activity using convergent cross-mapping. Our work presents four main innovations. First, we expound on the conceptual innovation that spontaneous activity of a local cortical ensemble in alert monkeys encodes the causal properties of a cortical circuit and can be used to predict manipulation effects at a granular spatiotemporal level. Our demonstration in array recordings from alert monkeys highlights that this approach is directly transferable to human subjects, where such devices have been used for decades, thus bearing important implications for future translational studies. Second, we introduce the new metric of interventional connectivity to characterize the effects of micro-stimulation on the spatial array geometry. Third, we introduce a methodological advancement showing how to estimate the statistical significance of causal flow inference using convergent cross-mapping, which is central to demonstrating the predictive power of causal flow. Fourth, we compare alternative approaches to estimate functional interactions from spontaneous activity and show that our causal flow approach performs significantly better than many commonly used alternative methods. Finally, we provide an open-source package to reproduce our results and encourage wide application of our approach in the neuroscience community and beyond. Our results establish a framework for discovering the rules that enable the generalization of resting state causal interactions to more complex behavioral states, paving the way toward targeted circuit manipulations in future brain-machine interfaces.

Micro-stimulation experiments have played a crucial role in our understanding of the organization and function of neural circuits in the primate brain. Among many successful examples are micro-stimulation of motion-selective middle temporal (MT) neurons to alter choice [1], reaction time [30], or confidence [31] of monkeys performing a direction discrimination task. Our approach to quantifying causal flow based on the activity of a large neural population spread out in multiple neighboring cortical columns provides an easily implementable solution with many advantages. First, we directly assess the network effects of the activity of each neuron, and thereby generate predictions about the impact that perturbing the activity of one electrode will have on the rest of the population. Second, population activity has proven quite powerful in revealing the neural computations that underlie behavior, with features that are robust to the exact identity of the recorded neurons [32–37].

Previous studies investigated the effects of electrical stimulation at the mesoscopic level in human and nonhuman primates. In human surgical patients, the spread of local electrical stimulations on local field potentials (LFP) across cortical areas could be predicted by using spontaneous activity data together with white matter connectivity [38]. In another study of epileptic patients, the effectiveness of seizure reduction in response to electrical stimulation could be predicted by functional connectivity measured from resting-state magnetoencephalography [39]. Changes in EEG frequencies induced by the stimulation at specific sites could be predicted from resting state EEG coherence [40]. The spatial location of stimulation-evoked potentials could be predicted from the resting state fMRI spectrum seeded at the stimulation site [41]. Alternative approaches aimed at predicting the effects of stimulation using network control theory, although at the whole brain level [42]. In nonhuman primates, a recent mesoscopic approach modeled the effect of time-varying electrical stimulations on LFP across cortical areas [43]. In this study, the effects of orbitofrontal cortex stimulation on the LFP activity of other areas could be predicted by using LFP activity at rest. These studies were based on linear methods [38,39,43], which performed well at the mesoscopic level. While these studies addressed the relationship between stimulation and spontaneous activity at the mesoscopic level, in our study, by contrast, we examined the effect of micro-stimulation on the spiking activity of a local cortical circuit on a single array geometry. We found that nonlinear time series forecasting methods (convergent cross-mapping) outperformed several alternative methods based on information theory (Granger Causality and related methods) for predicting stimulation effects based solely on spontaneous activity data. It would be interesting to compare mesoscopic methods [38,43] to our causal flow approach and we leave this direction to future work.

Previous studies characterized the effect of perturbations on single neurons in cortical circuits [44] and clarified the structure of anatomical circuit connectivity required to produce the observed effects [45]. In a similar fashion, we defined interventional connectivity as the effect of the perturbation of a single node on the activity of all other observed nodes. The uniqueness of our approach, compared to earlier studies, is the ability to predict the interventional connectivity from the causal flow inferred at rest. We suggest that characterizing causal flow during a task and using our model-based approach to predict the impact of a variety of perturbations on the population-level representations offer an attractive alternative to the traditional trial-and-error approaches where different neural clusters are manipulated in search of a desirable behavioral effect. We speculate that our model-based approach may lead to crucial advances in brain-machine interfaces if one can use causal flow inferred from resting activity to predict perturbation effects during a task—a direction we will actively pursue in the future (see [43] for a related approach).

Two key challenges in the interpretation of typical micro-stimulation experiments are (1) indirect activation of distant neurons through the activation of the neural cluster around the stimulating electrode, and (2) effects on fibers of passage that could cause direct activation of neurons distant to the stimulating electrode [46]. Our approach directly addresses the first challenge by mapping the causal flow based on the ensemble activity. The second effect acts as noise in our approach because the causal flow is quantified based only on the activity of the neurons recorded by the electrodes. The success of our approach (Fig. 3) suggests that this noise is not overwhelming. The robustness of our approach likely stems from the synergy of our delay embedding methods with our focus on the population neural responses and the large number of simultaneously recorded neural clusters in our experiments, which effectively capture key features of the intrinsic connectivity in the circuit (Fig. 1) [21,47–50]. A recent theoretical study [45] supports the viability of using biologically plausible models of cortical circuits in terms of E-I networks with structured connectivity to explain perturbation experiments in awake animals, although they do not attempt to predict the perturbation effects.

Current methods for establishing site efficacy for perturbation experiments are labor-intensive, time-consuming, and often unable to generalize beyond the limited task set they are optimized for. Although recent studies showed that one can predict the mesoscopic effects of electrical stimulation using measures of functional connectivity between cortical areas [38,43], here we focused on local stimulation effects between spiking activity on the same electrode array. We demonstrated a statistical method capable of predicting the impacts and efficacy of a targeted micro-stimulation site using only resting activity. Crucially, our method can directly be applied to monkeys and humans, where commonly used “large-scale” recording technologies often permit sampling from only a small fraction of neurons in a circuit (typically <1%). Our method is thus likely to improve the safety and duration of the procedure, a key step toward targeted circuit manipulations for ameliorating cognitive dysfunction in the human brain, as well as the development of future brain-machine interfaces.

Estimating functional connectivity is a formidable task, especially susceptible to errors in the presence of strong recurrent couplings, noise, and unobserved common inputs ubiquitous in cortical circuits. Even when unlimited data are available, sophisticated methods typically fail in the presence of strong correlations between unconnected neurons [51]. We defined functional interactions as the causal interaction of cortical neurons. Existing methods for estimating functional interactions between multidimensional time series include linear regression [52], Granger causality [53], and interareal coherence [54,55]. While correlation-based methods are problematic for weak correlations, entropy-based methods such as transfer entropy [56] require large amounts of data. Detecting a clear causal relationship by transfer entropy [56] or Granger causality [57–60] is not straightforward unless the system’s dynamical properties are well known. Further, confounding effects of phase delay [61], self-predictability in deterministic dynamics [11] or common inputs [62,63] limit the usefulness of Granger causality and transfer entropy. Alternatives such as inverse methods based on Ising models utilize time-consuming learning schemes [64] though recently faster algorithms have been proposed [65,66]. Other approaches applicable to spike trains include generalized linear models [67] or spike train cross-correlograms [68]. Remarkably, the latter method was successfully validated using optogenetic perturbations *in vivo*.

Inferring causal functional connectivity from *extremely sparse recordings* of neural activity is a long-standing problem. Our method relies on delay embedding techniques used for reconstructing nonlinear dynamical systems from their time series data with convergent cross-mapping [11]. Crucially, convergent cross-mapping was designed to work precisely in the sparse recording regime [12,13], where other methods fail. While this powerful framework has been successfully applied in ecology [11], and previously applied to ECoG data [16] and in neuronal cultures [15], here we pioneered its use for estimating causal functional connectivity from spiking activity of a neural population in awake monkeys between electrodes on the same array. Our results show that causal flow outperformed other information-based methods at the causality of interactions. An important innovation of our approach is the estimation of statistical significance of the reconstruction accuracy between pairs of time series, which was absent in previous studies [11,15,16]. Our significance estimation is based on the concept of twin-surrogate pairs, a surrogate data set that breaks causality while preserving the attractor topology [20]. The interventional connectivity prediction from causal flow at rest relied on the difference between significant and nonsignificant causal flow pairs.

We carried out a detailed comparison regarding whether intervention effects could be predicted via causal flow compared to information-based methods such as Granger causality and related methods. We found that the predictive ability of Granger causality was reasonable on simulated data from a continuous rate network (Fig. S11 [29]), where it was correlated with the effect of perturbations, although less than causal flow. However, neither Granger causality nor the other information-based methods were capable of predicting perturbation effects in awake monkeys. In the presence of strong recurrent couplings, complex nonlinear dynamics, common inputs, and sparse subsampling regimes, such as in cortical ensemble activity, the assumptions underlying Granger causality do not hold, leading to its expected failure to predict stimulation effects. On the other hand, the causal flow was designed to work precisely in the regime of cortical dynamics [11], and thus we expected it to perform well in all cases as confirmed by our results.

The traditional notion that resting, or spontaneous, activity, regarded as random noise or baseline, is devoid of useful information [69–71] has been recently challenged, suggesting that it may instead encapsulate fundamental features of a neural circuits’ functional architecture [21,72,73], providing a repertoire of network patterns of activation [49,74,75]. Background activity, quantified with local field potentials [76,77], single-neuron membrane potentials [78], or population spiking activity [27] encodes information about the animal’s behavioral state, including even fine-grained movements [79–81]. The population coupling of single neurons estimated during the resting periods *in vivo* is correlated with the synaptic input connection probability measured *in vitro* from the same cortical circuit [82]. In neuronal cultures, the causal functional connectivity inferred from ongoing activity is predictive of the structural connectivity estimated from electrical stimulation: functionally downstream neurons have faster response latency to stimulation compared to functionally upstream neurons [15]. In human and nonhuman primates, resting activity could be used to predict the mesoscopic effect of stimulations on the LFP power spectrum between multiple cortical areas [43] and, to some degree, the responsiveness to epileptic seizure treatment [38]. Our results extend these studies showing that spontaneous activity encodes the causality structure within a single cortical microcircuit in awake primates. Moreover, the comparison between information-based methods and causal flow suggests that the seemingly stochastic features of spontaneous activity may not reflect noise but instead encode fine-grained, deterministic features of high-dimensional network dynamics. Our results thus highlight the central role that spontaneous activity may play in revealing a circuit’s functional structure, potentially benefiting the design of manipulation experiments.

The estimation of functional causal flow as we presented it in this study is based on pairwise comparison between time series. In its current form, our method may not be directly generalized to detect triplets or higher-order interactions. An interesting direction for future work is the generalization of our method to capture multineuron perturbations, namely, predicting the joint causal flow between two source neurons and one target neuron. This generalization could pave the way to predicting the effect of simultaneous multi-electrode perturbation on the activity of downstream neurons. A powerful alternative method for modeling triplet interactions is the Partial Information Decomposition [83]. We hope to return to this question in future work.

Another potential limitation of causal flow stems from the fact that neuronal activity in frontal areas likely receives time-varying input from several other cortical and subcortical areas. Moreover, the recorded ensemble neurons may receive common inputs from unobserved neurons within the same local prefrontal circuit. These sources of external input may change across different periods of the resting state, due to changes in the animal’s internal state such as arousal levels. These contextual effects might present a potential challenge when generalizing causal flow predictions across different conditions (such as resting vs task-engaged sessions). Our results address this concern by showing that causal flow estimates are robust to both private noise and time-varying external inputs, typically encountered in cortical circuits (Fig. 4). It is an interesting open question to estimate how causal flow may generalize across different behavioral conditions, and we hope to report on this in the future.

## Supplementary Material

1
