# Supplementary material for: Predicting the effect of micro-stimulation on macaque prefrontal activity based on spontaneous circuit dynamics
Source: Phys Rev Res. Author manuscript; Available in PMC 2024 Dec 12. (PMC11636805; doi:10.1103/physrevresearch.5.043211)
Supplement: 1 [file NIHMS1995985-supplement-1.pdf]

SUPPLEMENTARY MATERIAL

Predicting the Effect of Micro-stimulation on Macaque Prefrontal Activity Based on Spontaneous Circuit Dynamics

A. Supplementary Methods

1. *Experimental data*

We recorded and perturbed the activity of neurons in the pre-arcuate gyrus (area 8Ar) of macaque monkeys (macaca mulatta) using chronically implanted multi-electrode Utah arrays (96 electrodes; Blackrock Microsystems). All experimental procedures conformed to the National Institutes of Health *Guide for the Care and Use of Laboratory Animals* and were approved by the New York University Animal Welfare Committee.

During the experiments, monkeys sat in a primate chair, with their heads fixed using a titanium head post. The monkey remained awake (open eyes) and rarely moved limbs during these resting blocks, which were typically 10-20 min long. We started a session with a resting period recording block that we used to quantify CFs and predict the perturbation effects. This block was followed with a recording and micro-stimulation block that we used for testing predictions about causal interactions.

Electrical micro-stimulation was delivered through individual electrodes of the Utah array. Micro-stimulation sites were chosen randomly. Micro-stimulation pulse trains consisted of low current (15 μA) biphasic pulses [1–3], each 0.2 ms long, delivered at 200 Hz. Pulse trains were 120 ms long and occurred once in any 5 s period. The exact time of the micro-stimulation with the 5s periods varied randomly. Electrophysiological recording was done in between the micro-stimulation trains.

Monkeys did not perform any task nor receive rewards. We monitored the monkey's eye and limb movements using infrared camera systems (Eyelink for eye tracking, 1 KHz sampling rate). The room was mildly lit and quiet. The electrodes of the Utah array were 1mm long with 400 μm spacing between adjacent electrodes, permitting simultaneous recordings from neighboring columns in a 4 mm $\times$ 4 mm region of cortex. Raw voltage signals were filtered and thresholded in real time to identify spikes. Spike waveforms and raw voltage were saved at 30 KHz sampling frequency for offline processing. After removal of electrical artifacts, all unsorted spike waveforms were retained for subsequent analysis. Because the arrays were chronically implanted, our recordings were stable during the session with no measurable change in the spike waveforms.

The electrophysiological recording was done between the micro-stimulation trains; it resumed with a short latency (<5 ms) at the end of each pulse train and continued until the beginning of the subsequent train. Micro-stimulation of the pre-arcuate gyrus with currents >50 μA could trigger saccadic eye movements [4]. Our low current micro-stimulation was chosen well below this motor threshold and never triggered saccades in our experiments.

2. *Convergent Cross-Mapping*

In contrast to more traditional causality-detection algorithms based on information transfer, which test the prediction of a downstream time series through information from an upstream one, CCM operates through "now-diction" (reconstruction of simultaneous time segments) of an upstream series through information from a downstream one. As ensured by a powerful theorem [5], the now-diction of the upstream channels tends toward zero error in the limit of infinite data size.

The high-dimensional time series constructed through embedding in (1) lives on a manifold diffeomorphic to the full attractor only as long as the embedding dimension d is larger than twice the dimensionality of the attractor [6], a statement that generalizes to the box-counting dimension for fractal attractors [5]. This can be far smaller than the number of variables involved in processing, as routinely happens in brain activity during any given task. The condition that d be large enough is sufficient for an ideal setting, but how to select d and τ for a real, noisy dataset has been the topic of a vast literature (see [7]), depending on the specifics of a dataset (see Fig. 7 for parameter optimization).

Our estimates were cross-validated by splitting each time series recorded during resting periods into two segments – a training period and a test period, comprising 90% and 10% of each resting block, respectively. Given two time series $x_i(t)$ and $x_j(t)$, d-dimensional time series of delay vectors $\boldsymbol{X}_i(t)$ and $\boldsymbol{X}_j(t)$ are constructed. To test the accuracy of reconstruction, the data are segmented into a training period and a test period. For each putative downstream vector, $\boldsymbol{X}_i(t)$ in the test sample, a reconstruction $\hat{\boldsymbol{X}}_i(t)$ is obtained by listing the k time points $t_l(t)$ $(l = 1, \dots, k)$ corresponding to the delay vectors that are the nearest neighbors to $\boldsymbol{X}_j(t)$ according to the euclidean distance $\Delta_l(t) = \|\boldsymbol{X}_j(t) - \boldsymbol{X}_j(t_l(t))\|$. For each neighbor, the corresponding weight is computed as a positive, decreasing function of its distance from $\boldsymbol{X}_j(t)$, namely $w_l(t) = f_l(\Delta_1(t), \dots, \Delta_k(t))$, normalized to yield the reconstruction

$$\hat{\boldsymbol{X}}_i(t) = \frac{\sum_{l=1}^{k} w_l(t)\boldsymbol{X}_j(t_l(t))}{\sum_{l=1}^{k} w_l(t)}$$

In the limit of infinitely long datasets, any finite k and

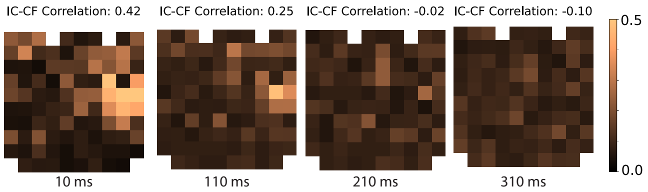

FIG. 6. **IC effects diminish through time in the monkey prefrontal cortex.** From left to right: IC of electrode 29 computed with $\Delta_{\text{post}} = 10, 110, 210, 310$ ms respectively.

any function f will yield asymptotically the same reconstruction. For a finite dataset, we adopted the common choice of uniform weight associated with the "simplex dimension" $k = d + 1$ [8]. The major computational bottleneck lies in the extraction of the nearest neighbors. We used a ball tree data structure, which partitions data in a series of nesting hyper-spheres as suitable to the structure of the training data.

Once reconstructions are obtained, their accuracy is estimated through the Pearson correlation coefficient ρ between the test-period time series $\boldsymbol{X}_i(t)$ and its reconstruction $\hat{\boldsymbol{X}}_i(t)$. In the noiseless infinite-data limit, such correlation saturates to one if a CF exists in the direction opposite to reconstruction. (Table III). Notice that the chosen measurement of reconstruction in terms of a Pearson correlation ρ is a random variable over the space of system trajectories but not necessarily a normal one as correlation coefficients are bounded between -1 and 1. This poses statistical problems to comparing different coefficients, and in particular coefficients in two converse directions ([9, 10]). To address this issue, we define the CF as the Fisher z-transform of the reconstruction coefficient ρ, which can be relied upon to have a near-normal distribution [11].

For the analyses of the ensemble spiking activity, we built delay vectors from spike counts with a delay time step equal to the bin width of 60ms, then z-score the spike counts.

3. *Estimating perturbation effects using IC*

We estimated perturbation effects by comparing network activity in intervals of length $T_{\max}$ ending Δ_{pre} before the onset and beginning Δ_{post} after the offset of each perturbation. Such periods reflected the transient pause in recording in those short periods adjacent to each micro-stimulation. The spiking activity of each target electrode was estimated in dT bins covering the lapse interval $T_{\max}$ and the distribution of spike counts was aggregated across all stimulation trials of the same source. The results were largely consistent for a variety of bin sizes and $T_{\max}$ values.

Assuming that the signals are stationary and given a sufficient number of trials, we denote the distribution of spike counts of electrode i prior to the stimulation of electrode j by $p(x_i|\texttt{no-stim}(x_j))$ and after the stimulation of electrode j by $p(x_i|\texttt{stim}(x_j))$. Using this notation, we define the stimulation effect of electrode j on electrode i as:

$$S_{ij} = \mathcal{D}\big(p(x_i|\texttt{no-stim}(x_j))|p(x_i|\texttt{stim}(x_j))\big)$$

where $\mathcal{D}$ is a divergence measure between distributions. This definition has roots in a subfield of statistics called causal inference, where the goal is to estimate the effect of a treatment (e.g. drug delivery) on an outcome (e.g. health indicator) while controlling for confounding factors (e.g. age or gender). Specific instantiations of $\mathcal{D}$ give rise to various IC measures. For example, if $\mathcal{D}(p(x)|q(x)) = \mathbb{E}_p[x] - \mathbb{E}_q[x]$ the resulting measure is known as average treatment effect (ATE) and various algorithms are developed for its estimation (e.g. propensity score matching [12]).

Here, we choose Kolmogorov-Smirnov (KS) distance between the pre and post-stimulation aggregated spike count distributions since it is non-parametric and is agnostic to the underlying data. KS test provides a normalized distance between 0 and 1 and a significance measure determining whether the stimulation of electrode j has a significant effect on the spike counts on electrode i (Fig. 1 and Fig. 3). The parameters used in our experiments are reported in Table II.

To further test the consistency of our estimator, we evaluated IC with $\Delta_{\text{post}} = 10, 110, 210, 310$ ms respectively and observed that the IC effects as well as correlations between IC and CF vanish when a sufficient amount of time is passed from the time of the stimulation (Fig. 6).

4. *Details of the twin surrogate method*

Each surrogate time series was constructed as the instantiation of a Markov process whose transition matrix has diagonal elements $p_{ii} = (n_i - 1)/n_i$, for n_i equal to the number of twins. This method has the advantage of preserving the temporal statistics of the full system, at the cost of introducing a hyperparameter, the neighborhood radius. We set the radius to be the tenth percentile of the distribution of the nearest-neighbor distances among Takens states. We defined the p-value for the significance of a CF value f as the fraction of twin-surrogate pairs that give a larger CF than f: $P\big(f(\text{surrogate}) > f\big)$.

When comparing the twin surrogate method to alternatives based on random shuffling of time points (which destroys all but the amplitude distribution) or isospectral surrogates (which preserves autocorrelations) we found that the latter two methods destroy not just the causal links but also any nonlinear property of the system (e.g., density distribution in phase space and its associated entropy), thereby increasing false positives in our statistical test [7].

5. *Partial correlation analysis for removing spatial dependence*

Both the CF and the perturbation effects decayed with distance from the source electrode. In order to control for this effect, we performed a partial correlation analysis by detrending the spatial dependence of CF and the perturbation effects using linear regression and then reevaluating the Pearson correlation between their residuals (Fig. 3H-I). Fixing a source j and given three measurements for each target i, namely $\boldsymbol{f}_j$ (CF), $\boldsymbol{s}_j$ (IC), and $\boldsymbol{d}_j$ (physical distance between all electrodes and j) we fit two linear regression models:

$$\boldsymbol{f}_j = \beta_0^{CF} + \beta_1^{CF}\boldsymbol{d}_j + \boldsymbol{\epsilon}_j^{CF}$$
$$\boldsymbol{s}_j = \beta_0^{IC} + \beta_1^{IC}\boldsymbol{d}_j + \boldsymbol{\epsilon}_j^{IC}$$

The partial correlation between CF and IC controlling for physical distance is then given by: $\rho(\boldsymbol{\epsilon}_j^{CF}, \boldsymbol{\epsilon}_j^{IC})$. This quantity allows us to measure the unique contribution of CF in predicting IC after removing their linear spatial dependence. We further repeated this experiment non-parametrically by subtracting the median curve shown in Fig. 3 and computing the correlation between the residuals. These results confirm our observation that the relationship between CF and KS is not simply a confound of the physical distance between the electrodes.

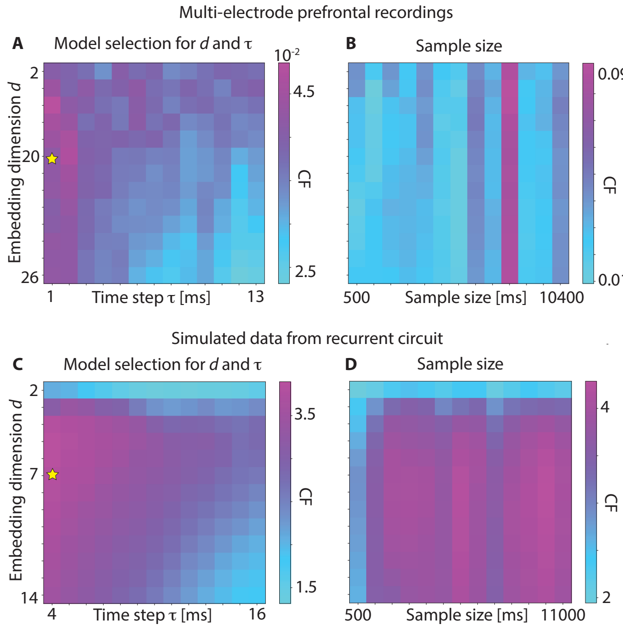

FIG. 7. **Model selection and sample size dependence.** The CF hyperparameters d (embedding dimension) and τ (time step) determining the delay vectors $\boldsymbol{X}_j = [x_j(t), j(t-\tau), \ldots, x_j(t - d\tau + \tau)]$ used for reconstruction were chosen by a hyperparameter search in the electrophysiological data from alert monkeys (A) and in the simulated continuous rate network (C). The asterisk indicates hyperparameters that maximize CF. The interaction between embedding dimension d and sample size used for CF inference in the electrophysiological data from alert monkeys (B) and in the simulated continuous rate network (D).

B. Supplementary Results

1. *Comparison to Other Causality Indices: Conceptual differences*

In the main text, we compared the performance of CF in predicting perturbation effects with that of several alternative methods based on information theory, including Granger causality in its univariate (GC) and multivariate versions (MGC); nonlinear GC (NGC) and extended GC (EGC), which perform autoregression using radial basis functions; and transfer entropy (TE). These methods rely on an assumption of “separability” between cause and effect, i.e., information on the upstream variable being transferred but not stored into the downstream variables. There are clear indications of this assumption breaking down in neurophysiological recordings [13]. This breakdown can occur even in the simplest systems. In panel A of Fig. 10, the 2D trajectory of an upstream variable (say, a network's activity) moves along a linear cycle with measurement noise on top. A downstream variable linearly integrates input from this upstream variable and is overlaid with extra measurement noise (see Methods for

Rate network simulations		
Parameter	Description	Value
g_r	Strength of the recurrent weights	4
g	Strength of $\mathcal{X}$ to $\mathcal{Y}$ weights	0.1
α	Rössler parameter	0.2
β	Rössler parameter	0.2
γ	Rössler parameter	5.7
λ	Recurrent leak term	1
τ_0	Time constant	1 ms

TABLE I. Parameters for the rate network.

Metadata and settings for stimulation response analysis		
Parameter	Description	Value
T_{resting}	duration of resting period recording	10 min
T_{pulse}	duration of stimulus	$\sim$ 120 ms
dT	time step used for response detection	7 ms
Δ_{pre}	time cushion before onset for extracting pre-pulse distributions	10 ms
Δ_{post}	time cushion after onset for extracting post-pulse distributions	4 ms
T_{max}	maximal time-lapse considered after stimulus	500 ms

TABLE II. Metadata and settings for stimulation response analysis.

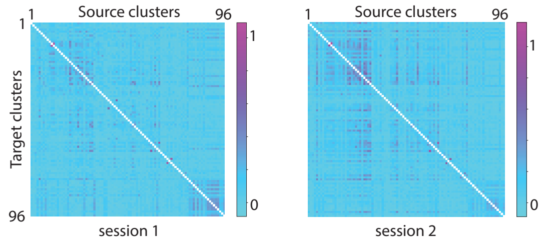

FIG. 8. **CF between electrodes in the monkey prefrontal cortex.** Full resting state CF matrix was inferred from ensemble spiking activity recorded by multi-electrode arrays in the pre-arcuate gyrus during quiet wakefulness. Two representative sessions for monkeys G and N are shown (left and right, respectively).

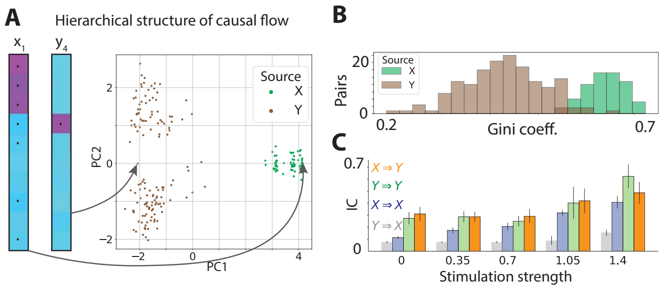

FIG. 9. **CF captures functional hierarchy in the simulated rate network.** A) The functional hierarchy in the network structure is encoded in the causal vectors. PCA of columns of the CF matrix. Each dot represents the causal vector of one source unit in a trial. Twenty trials with different initial conditions were simulated. The same $\mathcal{X}$ (green) and $\mathcal{Y}$ units (brown) of panel E are depicted. The separation of $\mathcal{X}$ and $\mathcal{Y}$ units is strongly preserved regardless of the initial condition. B) Distribution of Gini coefficient of causal vectors. C) IC is the Kolmogorov-Smirnov test statistics for the distribution of pre and post-stimulation activity of the target units. Left: IC matrix for 10 representative units. Black dots represent significant effects ($p < 0.05$). The effects of stimulating one source i on all targets k is encoded in the perturbation vector $\boldsymbol{s}^{(i)}$. Right: Perturbation effects increase with the stimulation strength S for source-target pairs of $\mathcal{X} \to \mathcal{X}$, $\mathcal{Y} \to \mathcal{Y}$, and $\mathcal{X} \to \mathcal{Y}$, but not $\mathcal{Y} \to \mathcal{X}$, reflecting the absence of feedback structural couplings from $\mathcal{Y}$ to $\mathcal{X}$. Error bars are s.e.m..

details). Application of GC – which assumes separability – detects causation in both directions, including the wrong one. By contrast, CF infers causality only in the correct direction.

The nested loop inside the trajectory of the downstream variable is a quintessential example of “downstream complexity” [14]. Because of that extra complexity, the mapping inference that CF attempts is less accurate in the downstream direction. GC, on the contrary, is concerned with detecting signal over noise and, regardless of the direction, is able to receive help from information in the source variable. Knowing the recent past of the downstream trajectory helps predict the next step of the upstream trajectory, which aside from the loop is equivalent.

GC also assumes monotonicity in causation trends, which cannot be expected to occur with real neurons. Consider the typical case of an external stimulus integrated linearly by a simple cell whose output is then integrated by a downstream complex cell with a nonlinear input-output function (Fig. 10, panel B; Methods for details). This basic architecture is already beyond the range of validity of Granger causality, which here detects no causality in either direction. By contrast, CF correctly

	CF	Feature
Upstream	$F_{ij} > 0$ and sig.; F_{ji} non-sig.	j is causally upstream of i.
Downstream	F_{ij} non-sig.; $F_{ji} > 0$ and sig.	j is causally downstream from i.
Reciprocal	$F_{ij} \sim F_{ji}$ and both sig.	i and j are reciprocally functionally connected.
Independent	F_{ij} and F_{ji} both non-sig.	i and j are causally independent.

TABLE III. Definitions and notations for the CF.

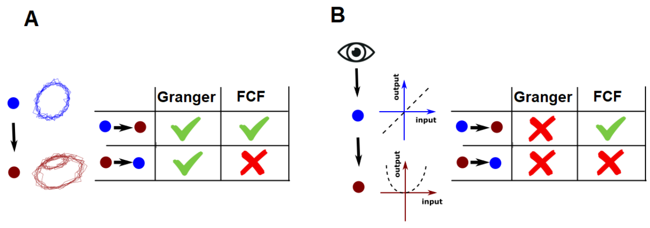

FIG. 10. **Conceptual comparison of Granger causality to CCM.** A) In the presence of nonseparable dynamics, the application of Granger causality detects causation in both directions, including the wrong one. By contrast, CCM infers causality only in the correct direction. B) Non-monotonic interactions as represented by a complex cell quadratically integrating signal from a simple cell. Granger causality fails in either direction. By contrast, CCM correctly infers causality only in the downstream direction. Overall, the absence of separability and monotonicity in a dataset can make the results of Granger causality unreliable.

infers causality only in the downstream direction.

2. *Comparison between CCM and Granger Causality*

We adopted the likelihood ratio as our Granger statistic. For the conceptual comparison in panel A of Fig. 10, we considered the straightforward solution to the following linear equations

$$\dot{x}_1 = -x_2 \quad (1)$$
$$\dot{x}_2 = x_1 \quad (2)$$
$$\dot{y}_1 = -2y_2 + x_2 \quad (3)$$
$$\dot{y}_2 = 2y_1 + x_2 \quad (4)$$

and added white zero-mean Gaussian measurement noise of standard deviation $\sigma = .1$ on top of all four variables. Causality between x_2 and y_2 was inferred with CCM-based CF and Granger Causality, using the same significance criterion ($p < 0.05$) for both directions and for both methods. Moreover, the same value $d = 10$ was used for both the maximal lag in Granger and the delay dimension for CCM (delay time $\tau = 1$).

In Fig. 10, panel B, the variable representing single-cell activity was taken to be the x-coordinate of a Lorenz attractor with parameters $\alpha = 10, \beta = 8/3, \rho = 28$, while the activity of the complex cell downstream of it evolved as $\dot{X} = -X^2 + x$. Causality was inferred with corresponding parameters in the two methods, exactly as done for panel A.

3. *Comparison to Other Causality Indices: Results*

A recent paper [15] investigates how different causality indices recover the direction of causation in simulations where there is a clear unidirectional influence from one variable onto the other. Below we first briefly explain each causality index. Then, we present results on the simulated rate network from Fig. 3 and monkey prefrontal data from Fig. 1 to investigate which indices can predict the perturbation effects measured by IC.

A general principle underlying all information-based causality indices is that causality is defined by the precedence of influence in time. If the past of variable X contains information about or allows the prediction of the future of variable Y then there is causal influence from X to Y. This is precisely the idea behind the definition of *Granger Causality (GC)* and its variants. If we assume that two signals evolve jointly according to an autoregressive model, then GC measures if the past of X, Y together helps predict the future of Y better than the past of Y alone. The significance test is performed using F-test as commonly done in the GC literature.

Transfer Entropy (TE) is defined similarly, but TE relaxes the autoregressive assumption to arbitrary rules for the stochastic evolution of time series, computing the conditional mutual information between the future of Y and past of X conditioned on the past of Y [16, 17]. It is worth noting that if the data follows an autoregressive model, GC and TE become equivalent. Although TE is nonparametric, its estimation is a challenging statistical task often requiring large amounts of data. For TE, here we use an estimator developed by [18] and employed by [15] which is based on nearest neighbor methods.

Although GC was originally developed for univariate and autoregressive signals, one can generalize it to multivariate and nonlinear counterparts. *Multivariate GC* (MGC) computes the same criterion as GC with the difference that the conditioning is done on all the other variables in the multivariate time series. This allows for measuring the unique predictability of the future of Y from the past of X when we control for other intermediate signals in the network. *Nonlinear GC* (NGC) performs the autoregression using radial basis functions [19]. *Extended GC* (EGC) provides another generalization to GC based on locally linear approximation [20].

Notice that in contrast to these methods which rely on the stochastic fluctuations of signals, CCM is based on the deterministic aspects of a dynamical system and instead of measuring noise statistics it uses nearest neighbors in the state space of a stationary dynamical system

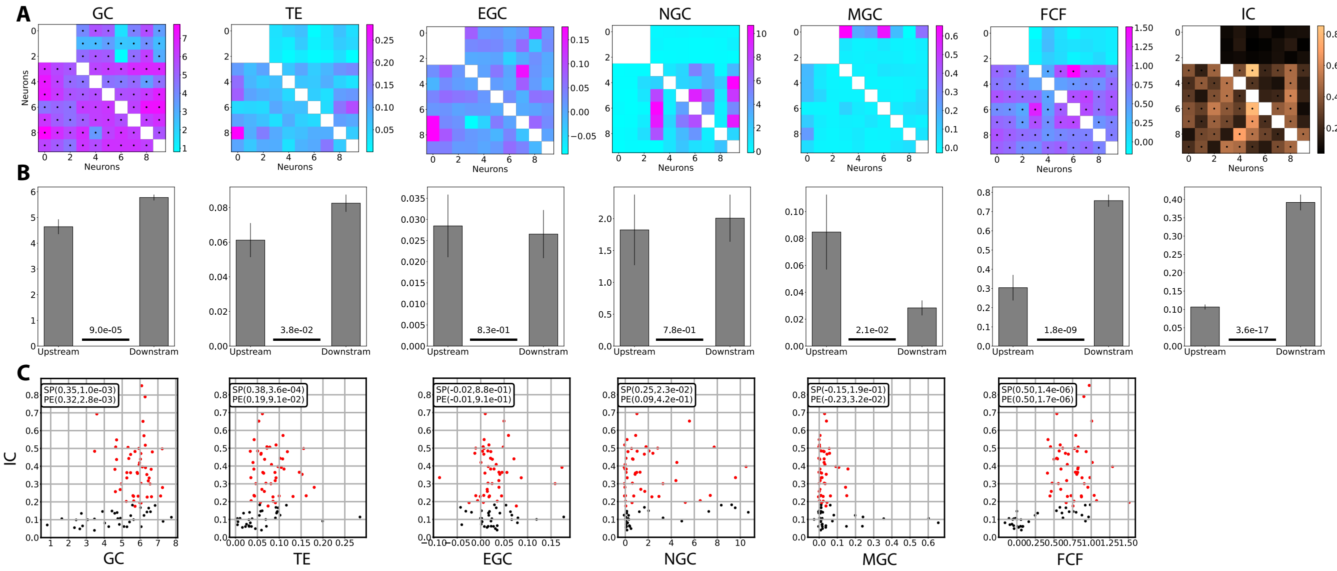

FIG. 11. **Comparison of causality indices on the simulated rate network.** A) Different causality indices applied to the simulated rate network of Fig. 3; from left: Granger Causality (GC), Transfer Entropy (TE), Extended Granger Causality (EGC), Nonlinear Granger Causality (NGC), Multivariate Granger Causality (MGC), CF, IC. B) Each bar plot corresponds to the causality index values separated according to the significance of the IC matrix (t-test with respective p-values reported); CF best reflects upstream vs. downstream as defined by the significant elements of IC. The rightmost bar plot corresponds to the IC values separated by their significance, providing a ceiling for the causality indices. C) Scatter plots of correlations between measured causality index on the x-axis and IC values on the y-axis (SP, PE represent Spearman and Pearson correlation coefficients and p-values, respectively). In this simulation, CF can best predict IC among the indices.

to predict one signal from the other. Moreover, in the limit of large datasets, CCM is less affected by the unobserved nodes due to the topological correspondence between the time-lagged history of each variable and the high-dimensional data-generating system which includes the unobserved nodes.

We summarize the results obtained from different causality indices in Fig. 6 and 1. In the simulated rate network, CCM and GC show significant positive correlations with IC, and CCM performs best. For the monkey data, CCM shows positive correlations with IC, whereas GC indices are not robust and generally underperform. TE fails perhaps due to the small sample size or the presence of observed and unobserved nodes in the network which are not accounted for. A summary of the hyperparameters used for the simulations and calculating different causality indices are included in the corresponding `config` file on the code repository released with this paper:
`https://github.com/amin-nejat/FCF/tree/master/example_configs`.

[1] C. D. Salzman, C. M. Murasugi, K. H. Britten, and W. T. Newsome, Microstimulation in visual area mt: effects on direction discrimination performance, Journal of Neuroscience **12**, 2331 (1992).

[2] S.-R. Afraz, R. Kiani, and H. Esteky, Microstimulation of inferotemporal cortex influences face categorization, Nature **442**, 692 (2006).

[3] C. R. Fetsch, R. Kiani, W. T. Newsome, and M. N. Shadlen, Effects of cortical microstimulation on confidence in a perceptual decision, Neuron **83**, 797 (2014).

[4] C. J. Bruce, M. E. Goldberg, M. C. Bushnell, and G. B. Stanton, Primate frontal eye fields. II. Physiological and anatomical correlates of electrically evoked eye movements, J Neurophysiol **54**, 714 (1985).

[5] T. Sauer, J. A. Yorke, and M. Casdagli, Embedology (1991).

[6] F. Takens, Detecting strange attractors in turbulence (1981).

[7] M. Thiel, M. C. Romano, J. Kurths, M. Rolfs, and R. Kliegl, Twin surrogates to test for complex synchronisation, EPL (Europhysics Letters) **75**, 535 (2006).

[8] G. Sugihara and R. M. May, Nonlinear forecasting as a way of distinguishing chaos from measurement error in time series, Nature **344**, 734 (1990).

[9] X. Wang, S. Piao, P. Ciais, P. Friedlingstein, R. B. Myneni, P. Cox, M. Heimann, J. Miller, S. Peng, T. Wang, *et al.*, A two-fold increase of carbon cycle sensitivity to tropical temperature variations, Nature **506**, 212 (2014).

[10] E. R. Deyle, M. Fogarty, C.-h. Hsieh, L. Kaufman, A. D. MacCall, S. B. Munch, C. T. Perretti, H. Ye, and G. Sugihara, Predicting climate effects on pacific sardine, Proceedings of the National Academy of Sciences **110**, 6430 (2013), https://www.pnas.org/content/110/16/6430.full.pdf.

[11] R. A. Fisher, *Statistical Methods for Research Workers* (Oliver and Boyd, Edinburgh, 1925) pp. ix + 239.

[12] P. R. Rosenbaum and D. B. Rubin, The central role of the propensity score in observational studies for causal effects, Biometrika **70**, 41 (1983).

[13] A. T. Baria, M. N. Baliki, T. Parrish, and A. V. Apkarian, Anatomical and functional assemblies of brain bold oscillations, Journal of Neuroscience **31**, 7910 (2011).

[14] S. Tajima, T. Yanagawa, N. Fujii, and T. Toyoizumi, Untangling Brain-Wide dynamics in consciousness by Cross-Embedding, PLoS Comput. Biol. **11**, e1004537 (2015).

[15] T. Edinburgh, S. J. Eglen, and A. Ercole, Causality indices for bivariate time series data: a comparative review of performance, Chaos: An Interdisciplinary Journal of Nonlinear Science **31**, 083111 (2021).

[16] T. Schreiber, Measuring information transfer, Physical review letters **85**, 461 (2000).

[17] R. Marschinski and H. Kantz, Analysing the information flow between financial time series, The European Physical Journal B-Condensed Matter and Complex Systems **30**, 275 (2002).

[18] A. Kraskov, H. Stögbauer, and P. Grassberger, Estimating mutual information, Physical review E **69**, 066138 (2004).

[19] N. Ancona, D. Marinazzo, and S. Stramaglia, Radial basis function approach to nonlinear granger causality of time series, Physical Review E **70**, 056221 (2004).

[20] Y. Chen, G. Rangarajan, J. Feng, and M. Ding, Analyzing multiple nonlinear time series with extended granger causality, Physics letters A **324**, 26 (2004).
